# Prolonged intermittent fever and massive splenomegaly in a miner working in the tropical jungle, China

**DOI:** 10.1371/journal.pntd.0008278

**Published:** 2020-07-09

**Authors:** Yanbin Liu, Zhiyong Zong

**Affiliations:** Center of Infectious Diseases, West China Hospital, Sichuan University, Chengdu, China; University of Texas Medical Branch, UNITED STATES

## Abstract

Prolonged fever is a particular challenge. A 47-year-old man with 5-year intermittent fever and remarkable splenomegaly was diagnosed as chronic melioidosis after splenectomy. The case would help clinicians to raise awareness and include chronic melioidosis in the differential diagnosis for patients with the travel history in melioidosis endemic regions.

## Presentation of case

**Fig 1:**
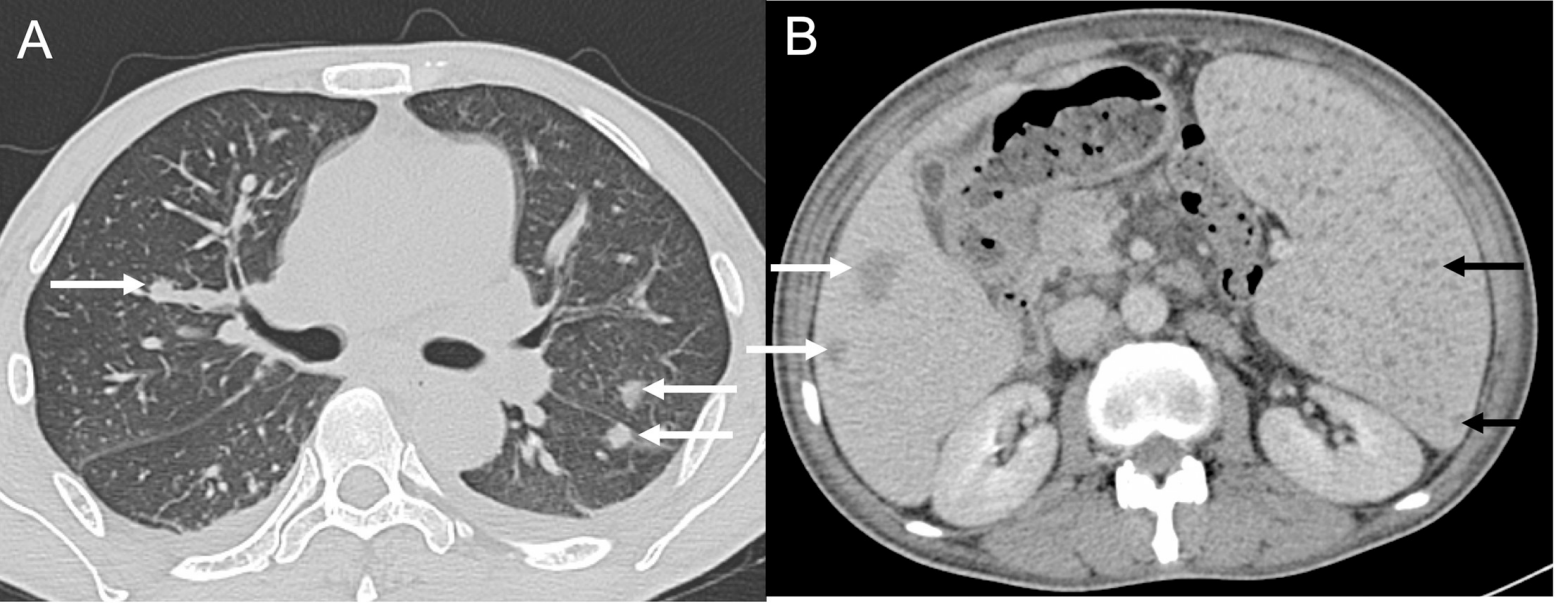
Clinical images from the patient. (A). An image of abdominal CT. Lesions of the liver are shown by white arrows, and nodes of spleen are shown by black arrows. (B). Spleen that was removed from the patient. Nodes in yellow are pointed out by arrows. CT, computed tomography.

A 47-year-old man was admitted with intermittent fever for five years. For the preceding 20 years, he had worked in the tropical jungle in Hainan Island, China as a gold miner. He had no history of diabetes and had normal fasting and postprandial plasma glucose levels on admission and in multiple subsequent tests. He also had no history of any other underlying diseases. On admission, his temperature was 39.5°C, and a blood test revealed a white blood cell count of 2.53 × 10^9^/L with 62.6% neutrophils, 28.1% lymphocytes, and 8.5% monocytes, hemoglobin of 4.9 g/dL, and platelets of 42 × 10^9^/L. Abdominal computed tomography revealed hepatomegaly, splenomegaly, multiple lesions of low density in the liver and spleen, enlarged lymph nodes at multiple sites, and ascites (Fig 1A). Two sets of blood cultures and one bone marrow culture grew no microorganisms using the BacT/ALERT 3D system (bioMérieux; Marcy l'Etoile, France). Liver biopsy guided by ultrasound revealed necrosis and chronic inflammation without microorganisms by Ziehl–Neelsen acid-fast, methenamine silver, and Periodic acid–Schiff staining. Culture of the liver biopsy sample also grew no microorganisms. The patient received a splenectomy. The spleen was around 25 cm in length with numerous 0.1 cm to 0.5 cm yellow nodules (Fig 1B). Spleen biopsy revealed chronic granulomatous inflammation and necrosis in the absence of microorganisms by Ziehl–Neelsen acid-fast, methenamine silver, and Periodic acid–Schiff staining. Culture of a spleen biopsy sample on a blood agar plate grew gram-negative bacilli, identified as *Burkholderia pseudomallei* by the Vitek 2 Compact automated system (bioMérieux) and matrix-assisted laser desorption ionization time of flight mass spectrum (MALDI-TOF; Bruker, Billerica, MA; with a 1.978 log[score], close to the greater than 2.0 log[score] to indicate a high confidence of identification). Species identification of the strain was further established by amplification and subsequent sequencing of the 16S rRNA gene with universal primers 27F/1492R [1] and the housekeeping *gyrB* gene (encoding DNA gyrase unit B), as described previously [2]. The 1,147-bp *gyrB* sequence and 1,345 bp nearly complete 16S rRNA gene sequence of the strain were 100% and 99.9% (1,344/1,345 bp) identical to those of *B*. *pseudomallei–*type strain ATCC 23343 (GenBank accession no. CWJA00000000.1). The identity of the strain was thus established as *B*. *pseudomallei*, confirming a diagnosis of chronic melioidosis. Multilocus-sequence typing (MLST) of seven housekeeping genes, as described previously [3], identified the strain as belonging to sequence type 55 (ST55), one of the dominant types in Hainan [4].

In vitro antimicrobial susceptibility testing was performed using Vitek 2. The strain was susceptible to imipenem, meropenem, levofloxacin, tetracycline, and trimethoprim/sulfamethoxazole. The patient became afebrile after splenectomy, and he received intravenous imipenem (500 mg three times daily) and intravenous levofloxacin (500 mg once daily) for two weeks, followed by oral levofloxacin (500 mg once daily) and oral trimethoprim/sulfamethoxazole (240 mg/1200 mg twice daily, which met the recommended dosage [5] in light of his 55 kg body weight) for three months. Trimethoprim/sulfamethoxazole is the first-line drug for the eradication phase of treatment for melioidosis [6], and current evidence suggests that oral trimethoprim/sulfamethoxazole alone is adequate for the eradication phase of treatment [7]. In view of the long clinical history, we considered that the patient might need enhanced treatment to eradicate *B*. *pseudomallei*, a facultative intracellular pathogen. We therefore added levofloxacin on the basis of in vitro susceptibility of the isolate and the known intracellular penetration of the agent. However, we acknowledge that the addition of levofloxacin may be unnecessary and should not be generalized. He remains well during the 6-month ongoing follow-up. The patient in this manuscript provided written informed consent (as outlined in the PLOS consent form) to the publication of the case details.

## Case discussion

### The diagnosis of this case

This patient had low white blood cell count and low hemoglobin level, which could be explained by the presence of the massive splenomegaly. The prolonged disease may also lead to reduced hemoglobin level. Of note, this patient had multiple lesions in the liver, spleen, and lungs and prolonged intermittent high fever, raising the possibility of disseminated infections of pathogens with low virulence (e.g., tuberculosis, nocardiosis, and cryptococcosis) or malignancies as the diagnosis. He had no known immunosuppressing conditions, making nocardiosis and cryptococcosis less likely in this case. An interferon-γ release assay for tuberculosis (Wantai BioPharm; Beijing, China) was negative. The negative interferon-γ release assay and the absence of acid-fast bacilli in biopsies did not support the diagnosis of tuberculosis. Most of mainland China is outside the range of *B*. *pseudomallei* endemicity, but melioidosis is endemic in Hainan Island in southern China [8, 9]. We therefore considered the possibility of melioidosis in this patient. The growth of *B*. *pseudomallei* from the spleen biopsy sample established the diagnosis of melioidosis. Selective culture of a throat swab or urine sample may have helped to make an accurate diagnosis [10], but unfortunately such culture is not available in our hospital.

### Chronic melioidosis and diagnosis

Melioidosis can present as both acute and chronic illness with varied symptoms, making it difficult to establish the correct diagnosis [11]. Chronic melioidosis is defined by illness with symptoms for longer than two months [12, 13]. Chronic melioidosis is relatively uncommon and occurs in about 8% to 12% of melioidosis cases in endemic regions, including Thailand and Northern Australia [12, 14], and in 4.1% in Hainan Island [9]. Prolonged fever of unknown origin is a particular challenge for clinical management and can be caused by a variety of pathogens and noninfectious diseases, e.g., malignancy [15]. As a result of human movement, diseases can be seen in locations where they are not consistently endemic, imposing additional difficulties for diagnosis. We believe that the case reported here will help clinicians to recognize chronic melioidosis, which could cause prolonged fever for years and massive splenomegaly, and to include this disease in the differential diagnosis on fever of unknown origin for patients who had history of travel to, or resident in, melioidosis endemic regions. Although melioidosis is well known in Southeast Asia and Northern Australia, China is largely neglected. This case also highlights the urgent need for studying melioidosis in sporadically endemic regions other than Southeast Asia and Northern Australia, such as southern China.

**Key learning points**

- Melioidosis can be missed by clinicians in nonendemic regions.
- Chronic melioidosis can lead to prolonged fever for years and massive splenomegaly.
- Melioidosis should be included in the differential diagnosis of fever of unknown origin for patients who have travel or resident history in melioidosis sporadically endemic regions such as Hainan Island, China.
- Culture of biopsies may help confirm the diagnosis of chronic melioidosis.
